# Efficacy of tranexamic acid in reducing blood loss in posterior lumbar spine surgery for degenerative spinal stenosis with instability: a retrospective case control study

**DOI:** 10.1186/1471-2482-11-29

**Published:** 2011-11-03

**Authors:** Stefan Endres, Martin Heinz, Axel Wilke

**Affiliations:** 1 Department of Orthopaedic Surgery, Elisabeth-Klinik Bigge/Olsberg, Heinrich-Sommer-Str. 4, 59939 Olsberg, Germany

## Abstract

**Background:**

Degenerative spinal stenosis and instability requiring multilevel spine surgery has been associated with large blood losses. Factors that affect perioperative blood loss include time of surgery, surgical procedure, patient height, combined anterior/posterior approaches, number of levels fused, blood salvage techniques, and the use of anti-fibrinolytic medications. This study was done to evaluate the efficacy of tranexamic acid in reducing blood loss in spine surgery.

**Methods:**

This retrospective case control study includes 97 patients who had to undergo surgery because of degenerative lumbar spinal stenosis and instability. All operations included spinal decompression, interbody fusion and posterior instrumentation (4-5 segments). Forty-six patients received 1 g tranexamic acid intravenous, preoperative and six hours and twelve hours postoperative; 51 patients without tranexamic acid administration were evaluated as a control group. Based on the records, the intra- and postoperative blood losses were measured by evaluating the drainage and cell saver systems 6, 12 and 24 hours post operation. Additionally, hemoglobin concentration and platelet concentration were reviewed. Furthermore, the number of red cell transfusions given and complications associated with tranexamic acid were assessed.

**Results:**

The postoperative hemoglobin concentration demonstrated a statistically significant difference with a p value of 0.0130 showing superiority for tranexamic acid use (tranexamic acid group: 11.08 g/dl, SD: 1.68; control group: 10.29 g/dl, SD: 1.39). The intraoperative cell saver volume and drainage volume after 24 h demonstrated a significant difference as well, which indicates a less blood loss in the tranexamic acid group than the control group. The postoperative drainage volume at12 hours showed no significant differences; nor did the platelet concentration Allogenic blood transfusion (two red cell units) was needed for eight patients in the tranexamic acid group and nine in the control group because of postoperative anemia. Complications associated with the administration of tranexamic acid, e.g. renal failure, deep vein thrombosis or pulmonary embolism did not occur.

**Conclusions:**

This study suggests a less blood loss when administering tranexamic acid in posterior lumbar spine surgery as demonstrated by the higher postoperative hemoglobin concentration and the less blood loss. But given the relatively small volume of blood loss in the patients of this study it is underpowered to show a difference in transfusion rates.

## Background

Degenerative spinal stenosis and instability requiring multilevel spine surgery can be associated with considerable blood loss. The association of increased intra- and postoperative blood loss during reconstructive spine surgery with higher complication rates has been established [1,2]. Typical consequences of blood loss are extended operation times and pulmonary and cerebral edema due to fluid shifts [2]. As a consequence, blood transfusions are often required. Yet blood transfusions are not free of risks, including transfer of infectious agents, increased risk of postoperative infections and immunological sensitizing including transfusion-related acute lung injury can occur [3,4]. Measures to decrease transfusion-related complications such as preoperative autologous blood donation, application of cell saver-systems or the use of erythropoietin are often associated with higher costs and logistic challenges [5-7].

More recently, the use of anti-fibrinolytics has come into favor for orthopedic surgery. Recent studies have shown that tranexamic acid is efficient in reducing blood loss in orthopedic surgery [8-10]. Considering the risks associated with allogenic blood transfusions, we aimed in this study to evaluate the efficacy of tranexamic acid in reducing blood loss and the need for allogenic blood transfusion in patients undergoing posterior lumbar spine surgery. Additionally, we observed the appearance or absence of perioperative complications that may be associated with the use of tranexamic acid.

## Methods

### Study Design

Between January 2009 and December 2010, we enrolled 97 patients who were to have a posterior lumbar spine surgery in this retrospective case control study. All patients were in need of spinal fusion surgery of 4 to 5 segments owing to degenerative spinal stenosis with instability. Exclusion criteria were renal dysfunction identified by a glomerular filtration rate lower than 50 ml/min, current use of anti-coagulant medication, any history of coronary artery disease with stent placement and history of thromboembolic events.

The surgery and follow up were performed by the authors (S.E. and M.H.). All patients underwent fusion with pedicle screws and rod instrumentation (Tango RS, Fa. Ulrich, Germany) and intervertebral fusion (PLIF - Prospace Aesculap, porous titanium). Posterior lumbar interbody fusion was performed over one to three levels and in all cases a posterolateral bone graft was done. The bone graft for fusion (posterolateral fusion) was a mixture of Endobone^® ^(Biomet, Germany)and autologous bone obtained from the decompression procedure. No iliac crest bone graft harvesting was performed. Laminectomy, partial resection of the facet and a foraminotomy were performed on all patients over at least 3 levels. The average number of posterior instrumented levels was 4.8 (range 4-5).

None of the patients had a disease of the coagulation system, no positive anamnesis of a DVT or a higher risk of bleeding. The two study groups were comparable in age, weight, height, sex and ASA physical status. The preoperative coagulation parameters, hemoglobin value and platelet count were within normal range and showed no significant differences between groups. The control group had undergone the lumbar spinal fusion procedure in 2009 before tranexamic acid use was introduced into our institution and consisted of 51 patients. The tranexamic acid group included 46 patients, enrolled in 2010, who received 1 g tranexamic acid intravenous preoperative (60 min before surgery) and six hours and twelve hours postoperative, based on the study by Zohar et al. [11].

On the basis of the records, the intraoperative (cell saver drainage volume) and postoperative (self-suction device) blood losses were measured by evaluating the drainage and cell saver systems 6, 12 and 24 hours postoperatively. Cell saver systems were used in all cases. Additionally, hemoglobin concentration and thrombocyte counts were reviewed. Furthermore, the numbers of red cell transfusions were assessed, and complications such as suspected medication or allergic reaction, suspected myocardial infarction, stroke, deep vein thrombosis, pulmonary embolism and renal failure were analyzed.

### Ethical board statement

Approval for the current study was given by the ethical board of the University of Münster, Germany [AZ 2010-218-f-s]. This project was performed in accordance with the Helsinki Declaration and with local legislation.

### Statistical Analysis

The data were analyzed using SPSS software (version 10.0; SPSS, Chicago, IL). Results are presented as mean ± SD. The independent Student's t test was used to compare the two groups. Differences were considered significant if the p-value was ≤ 0.05.

Chi quadrat testing was done to compare proportion between two groups, e.g. ASA classification and surgical procedures (PLIF and number of segments).

## Results

The established practice in our department is that patients are transfused if postoperative hemoglobin is < 8 mg/dl in patients with no coronary heart disease, or < 10 mg/dl in patients who have coronary heart disease and physiological signs of inadequate oxygenation.

The patient population consisted of 46 in the tranexamic acid group and 51 in the control group. The tranexamic acid group comprised 31 males and 15 females, mean age 67 years (SD: 10.5); in the control group there were 27 males and 24 females with a mean age of 69 years (SD: 9.8). Mean patient weights were 81.98 kg (SD: 14.53) for the tranexamic acid and 79.78 kg (SD: 13.99) for the control group. The duration of surgery was 172.74 min (SD: 41.58) for the tranexamic acid group and 168.09 min (SD: 42.03) for the control group. A review of the anesthesia records for the patients showed that 13 patients were of ASA class I, 21 of ASA class II and 12 of ASA class III in the tranexamic acid group; and nine were of ASA class I, 16 of ASA class II and 26 of ASA class III in the control group (Table 1).

**Table 1:** Demographics

	tranexamic acid group	SD	control group	SD	p value
**Number of patients**	46		51		
**Sex (M/F)**	31/15		27/24		
**Weight (kg)**	81.98	14.53	79.78	13.99	0.449
**Height (cm)**	168.00	6.77	171.35	7.47	0.023
**Age**	67	10.5	69	9.8	0.334
**PTT (s)**	27	3.93	26	4.2	0.230
					
**Duration of operation (min)**	172.74	41.85	168.09	42.03	0.587
					
**Surgical procedure**					0.286
1 segment PLIF	9		17		
2 segments PLIF	23		23		
3 segments PLIF	12		8		
4 segments PLIF	1		3		
5 segments PLIF	1		0		
					
**Surgical procedure**					0.308
1 segment instrumentation	0		0		
2 segments instrumentation	0		0		
3 segments instrumentation	0		0		
4 segments instrumentation	11		8		
5 segments instrumentation	35		43		
					
**ASA I/II/III**	13/21/12		9/16/26		0.042

The observed intraoperative infused volume (2554.55 ml/SD: 737.26 ml in the tranexamic acid group versus 2433.33 ml/SD: 737.26 ml 782.07 ml in the control group) and surgical time (172.74 min/SD: 41.85 min in the tranexamic acid group versus 168.09 min/SD: 42.03 min in the control group) were similar with no statistically significant differences between the groups.

The mean drop in the postoperative hemoglobin concentration was 3.83 g/dl in the tranexamic acid group and 4.21 g/dl in the control group, a difference of 10.1%. This difference was statistically significant and showed that the tranexamic acid group was superior (tranexamic acid group: 11.08 g/dl, SD: 1.68; control group: 10.29 g/dl, SD: 1.39; (p = 0.013).

The intraoperative cell saver volume (tranexamic acid group: 470 ml, SD: 153.06; control group: 560 ml, SD: 67.59; p = 0.0002) and the postoperative drainage volume at 24 hours (tranexamic acid group: 270, SD: 180; control group: 368.75, SD: 211.4; p = 0.0156) also demonstrated a significant difference which indicates a less blood loss in the tranexamic acid group than the control group.

The postoperative drainage volumes at 12 hours showed no significant differences; nor did the platelet concentration or complications.

Allogenic blood transfusion (two red cell units) was given to eight patients in the tranexamic acid group and to nine patients in the control group because of postoperative anemia. Intraoperative blood loss was too low to obtain autologous blood in the cell saver systems. Therefore no autologous blood transfusions were given in either group.

No complications associated with the administration of tranexamic acid occurred, such as suspected medication or allergic reaction, myocardial infarction, stroke, renal failure, deep vein thrombosis or pulmonary embolism. Overall results are given in table 2.

**Table 2:** Postoperative data

	tranexamic acid group	SD	control group	SD	p value
**Number of patients**	46		51		
**Hemoglobin (g/dl)**					
pre operation	14.91	1.1	14.51	1.28	0.103
post operation 1 day	11.08	1.68	10.29	1.39	0.013
mean drop	3.83	0.89	4.21	0.9	0.039
					
**Platelet concentration (tsd/μl)**					
pre operation	256.75	63.18	268.92	75.71	0.395
post operation 1 day	198.17	57.95	220.71	62.26	0.069
					
**Drainage (ml)**					
intra operation	470	153.06	560	67.59	0.0002
post operation 6 h	171.25	141.01	258.75	139.8	0.002
post operation 12 h	101.25	72.7	110	131.6	0.69
postop total 24 h	270	180	368.75	211.4	0.016
					
**intraoperative infusions (crystalloids in ml)**	2.554.55	737.26	2.433.33	782.07	0.435

## Discussion

Among patients undergoing major orthopedic and spine surgery, antifibrinolytic agents compared to placebo reduce bleeding, reduce the risk of transfusion and do not appear to increase the risk of myocardial infarction, stroke, deep vein thrombosis or pulmonary embolism [8,12-19]. These observations are consistent with those found in the cardiovascular surgery literature [20,21].

The aim of the present study was to evaluate the efficacy of tranexamic acid in reducing blood loss and the need for allogenic blood transfusion in patients undergoing posterior lumbar spine surgery. Our results suggest a less blood loss as shown by the higher postoperative hemoglobin concentration and lower cell saver volume in the tranexamic acid group. But we found no significant reduction of allogenic blood transfusion rate with the use of tranexamic acid. No adverse events with the use of tranexamic acid were seen in our population.

Heterogeneous patient populations (adolescent scoliosis, neuromuscular scoliosis, acquired degenerative instability, etc.), differences in surgical techniques (anterior, posterior, anterior-posterior, lumbar and cervical), heterogeneity in the doses and type of antifibrinolytic agent, timing of administration, and a lack of standardized dose regimens and transfusion thresholds may help to explain why the results of the present study are different from those previously published, which showed differences in blood loss and transfusion between patients who received tranexamic acid and those who did not.

Elwatidy et al. reported on the efficacy and safety of a large dose of tranexamic acid in spine surgery [9]. They enrolled 64 patients of whom 18 had multilevel anterior cervical discectomies, 22 had decompressive surgery for multisegmental stenosis, 15 had laminectomy and posterior instrumentation and nine had laminectomy and resection of a spinal tumor. The blood loss during surgery from patients in TA group was almost half the amount lost from patients in Placebo group (49% reduction). Consequently the amount of blood transfusion was 80% less in TA than in placebo group. The heterogeneity of this study population makes it difficult to compare the results because these procedures entailed more or less significant blood losses.

Another study by Baldus et al. compared the safety and efficacy of aprotinin and tranexamic acid in controlling blood loss during lumbar pedicle subtraction osteotomy (PSO) in adults [22]. The main difference from the present study is that the surgical procedure of PSO is substantially more complex (duration in the operating room: 7.5 hours versus 8.1 hours) and entails greater blood loss (mean 1114 ml - 2260 ml) than decompressive surgery with interbody fusion and instrumentation

Colomnia et al. performed a retrospective case control study to determine the impact of aprotinin or tranexamic acid use on reducing intraoperative blood loss and transfusion needs in complex spine surgery. They enrolled patients with diagnoses of adult scoliosis, neuromuscular scoliosis, congenital scoliosis, degenerative lumbosacral disease and posttraumatic kyphosis. The surgical procedures varied and included posterior instrumented fusion, anterior instrumented fusion, anterior plus posterior instrumented fusion, posterior lumbar interbody fusion (PLIF), pedicle subtraction osteotomy and Smith-Peterson osteotomy. The duration of surgery was 448 min, the numbers of levels fused 7.6 and the total blood loss was 1608 ml for tranexamic acid group. Therefore the total transfusion rate was 2.6. The authors found that the duration of surgery was the main predictive factor of total blood loss among the patients [23].

In conclusion, the present authors presume that the duration of the surgical procedure and type of surgery are predictive factors for significant blood loss and transfusion requirement in spine surgery. It is likely that tranexamic acid use results in a greater reduction in blood loss and transfusion the longer the surgical procedure lasts and therefore the greater the blood loss is.

Although we conducted a retrospective case-control study and the possibility of the results being affected by recall bias due to historical controls cannot be ruled out, the study provides evidence that the use of tranexamic acid in posterior lumbar spine surgery is not always necessary.

Given the small volume of blood loss in the patients, it seems that this study is underpowered to show a difference in transfusion rates. A much larger sample size would be necessary to prove this.

But the strengths of this study are the homogenous patient population in both groups, and the fact that the surgery was performed by a single surgeon.

## Conclusions

On the basis of this retrospective case control study, tranexamic acid use seems not to reduce transfusion rates in posterior lumbar spine surgery even if less blood loss was detectable. This could be explained by the lower bleeding risk and smaller overall blood loss of a posterior approach. Therefore the routine use of tranexamic acid in posterior spinal surgery has to be carefully considered to avoid unnecessary complications.

## Competing interests

The authors declare that they have no competing interests.

## Authors' contributions

SE designed the study. MH, AW and SE acquired, interpreted the data and wrote the manuscript. Statistics were done by SE and AW. All authors read and approved the final manuscript.

## Pre-publication history

The pre-publication history for this paper can be accessed here:

http://www.biomedcentral.com/1471-2482/11/29/prepub
